# Silencing of *ARL14* Gene Induces Lung Adenocarcinoma Cells to a Dormant State

**DOI:** 10.3389/fcell.2019.00238

**Published:** 2019-10-15

**Authors:** Fei Guo, Dexiao Yuan, Junling Zhang, Hang Zhang, Chen Wang, Lin Zhu, Jianghong Zhang, Yan Pan, Chunlin Shao

**Affiliations:** Institute of Radiation Medicine, Fudan University, Shanghai, China

**Keywords:** *ARL14*, *CIDEC*, ERK/p38, lung cancer dormancy, radiation

## Abstract

Recently, a growing number of ADP ribosylation factor (ARF) family members has been suggested to be critical in tumorigenesis. However, the effects of most ARF members on lung adenocarcinoma pathogenesis are still not well disclosed yet. In this study, *ARF-like GTPase 14 (ARL14)* was screened as an important prognostic factor of lung adenocarcinoma from The Cancer Genome Atlas (TCGA) database and validated by our *in vitro* experiments. It was found that silencing of *ARL14* gene inhibited cell proliferation and the abilities of cell migration and invasion, and it also attenuated radiation damage of lung adenocarcinoma cells but had no effect on the proliferation of normal lung cells. Notably, *ARL14* siRNA blocked the extracellular signal-regulated kinase (ERK)/p38 signaling pathway and induced cell cycle arrest in G0 phase, ultimately leading to cell dormancy. Moreover, *ARL14* siRNA enhanced the expression of *cell death activator DFFA-like effector* (*CIDEC*) that had opposite roles in cell proliferation and migration to *ALR14*. Collectively, our results suggest that *ARL14* has an important role in the pathogenesis of lung adenocarcinoma through CIDEC/ERK/p38 signaling pathway, and thus it could be applied as a new candidate of prognosis indicator and/or therapeutic target of lung adenocarcinoma.

## Introduction

With the development of diagnostic techniques and treatment strategies, significant improvements have been made in the quality of life of patients with lung cancer; however, malignant lung tumors still show the highest morbidity and mortality rates among cancer types (Siegel et al., 2017), with only 16.8% of patients surviving for 5 years after diagnosis (Ridge et al., 2013). Given the variability between pathology and etiology, lung cancer can be subdivided into non-small cell lung cancer (NSCLC) and small cell lung cancer (SCLC) (Sharma et al., 2007; Cheng et al., 2015). Approximately 40% of all lung cancer cases are lung adenocarcinoma, which is the most common type of NSCLC (Xue et al., 2018). The prognosis of patients with lung adenocarcinoma is extremely poor because of the lack of effective treatment measures against metastatic lung cancer. Therefore, studies to determine the molecular mechanisms governing the oncogenesis and metastasis process of lung adenocarcinoma are urgently needed.

*ADP ribosylation factor-like GTPase 14* (*ARL14*), also known as *ARF7*, belongs to the ADP ribosylation factor (ARF) family of GTP-binding proteins of the Ras superfamily and it is the closest homolog of *ARL11* (Yendamuri et al., 2007). The *ARL11* polymorphisms Trp149Stop and Cys148Arg have been shown to be associated with a high risk of familial cancers, such as breast, ovarian, colorectal, and hematological malignancies, among others (Calin et al., 2005; Frank et al., 2006; Masojc et al., 2006; Siltanen et al., 2008; Yang et al., 2009; Hamadou et al., 2017). *ARL11* was also reported as a novel tumor suppressor gene in lung and prostate cancer (Yendamuri et al., 2007, 2008; Siltanen et al., 2013). However, the function of *ARL14* in the formation and progression of human cancer is unknown.

This study was conducted to determine the function and possible underlying mechanisms of *ARL14* in lung adenocarcinoma tumorigenesis. Our results revealed the contribution of *ARL14* in lung adenocarcinoma tumorigenesis and suggested that *ARL14* might have potential implication as a diagnostic biomarker and therapeutic target for lung adenocarcinoma.

## Materials and Methods

### Cell Culture and Irradiation

Human lung bronchial epithelial BEAS-2B cells and human lung cancer PC9 cells were obtained as gifts from the Nanjing Medical University and School of Life Sciences of Fudan University, respectively. They were cultured in Dulbecco’s Modified Eagle Medium (DMEM). Human non-small-cell lung cancer A549 cells and human lung fibroblast MRC-5 cells were purchased from Shanghai Cell Bank (Shanghai, China) and cultured in DMEM and α-modified Eagle medium (MEM), respectively. All cells were cultured with suitable medium contained 10% fetal bovine serum (FBS, Gibco, Invitrogen, United States), 100 U/ml penicillin and 100 μg/ml streptomycin, and incubated at 37°C and 5% CO_2_ atmosphere. For irradiation treatment, cells were exposed to different doses of γ-rays as described previously (He et al., 2014).

### Transient Transfection of SiRNA

Short interfering RNAs (siRNAs) against *ARL14*, cell death activator DFFA-like effector (*CIDEC*) and their negative controls (RiboBio Biotechnology, Guangzhou, China) were transfected into cells with lipofectamine 2000 (Invitrogen, Carlsbad, CA, United States) following the manufacturer’s protocol. The target sequences of these transiently transfected siRNAs are listed in Supplementary Table S1.

### Cell Growth and Cloning Efficiency Assays

Following siRNA transfection for 24 h, 1000 cells/well were plated into a 96-well plate and incubated for 24, 48, 72, 96, or 120 h and then measured by Cell Counting Kit-8 assay (CCK-8, Dojindo Laboratories, Kumamoto, Japan) at an absorbance of 450 nm. For the cloning efficiency assay, 200–300 cells/well were seeded into six-well culture plates and grown for 10–14 days. The number of colonies containing more than 50 cells was counted and normalized to corresponding control.

### Radiation Sensitivity Assay

After siRNA transfection for 24 h, 1500–2500 cells/well from all groups were plated onto a 96-well plate and incubated for 24 h. After irradiation with γ-ray at 0, 2, 4, and 8 Gy, the cells were further cultured for 96 h and then issued for cell proliferation assay described above.

### Cell Migration and Invasion Assay

*In vitro* transwell assays were performed to assess cell migration and invasion abilities as previously described (Pan et al., 2016). Briefly, for the migration assays, 5–7 × 10^4^ serum-starved cells were cultured with serum-free medium in a upper insert dish containing enormous 8-μm-diameter pores in its bottom membrane (Corning Inc., Corning, NY, United States) companied with a 6-well plate chamber filled with DMEM containing 10% FBS. For the invasion assays, the above insert dish was replaced with one coated with 1 μg/mL Matrigel (Corning). After 24 h of culture, the cells were fixed with 100% methanol for 30 min and stained with crystal violet staining solution (Beyotime, Shanghai, China) for 25 min. Cells on the upper surface of the insert dish bottom were carefully removed using a wet cotton swab and those that had migrated through the membrane were photographed and counted in five random fields (×10) using an inverted microscope.

### Western Blot Assay

Western blot analysis for specific protein expression was performed as previously described (Wang et al., 2017). The antibodies used in this study are listed in Supplementary Table S2.

### Immunofluorescence Assay of Ki67 Protein

For all groups, 2–4 × 10^4^ cells plated on culture slides were incubated for 48 h at 37°C in 5% CO_2_, and then the exponentially growing cells were fixed with immune staining fix solution and treated with enhanced immunostaining permeabilization buffer for 15 min at room temperature. Next, non-specific antibody binding sites were blocked with QuickBlock^TM^ blocking buffer for immunological staining for 1 h. Ki67 primary antibody at appropriate dilutions was added and incubated at 4°C overnight followed by further incubation for 1 h at room temperature in the dark with Alexa Fluor^®^ 594 goat anti-mouse IgG (H + L) (Thermo Fisher Scientific, Waltham, MA, United States). Finally, the cell nuclei were counterstained with DAPI Fluromount-G^TM^ (Southern Biotech, Birmingham, AL, United States) for 5 min. The Ki67 positive cells were examined using a Zeiss Axioplan fluorescence microscope (Oberkochen, Germany).

### RNA Isolation and Quantitative Real-Time PCR Analysis

Total RNA was isolated from cells using a MiniBEST Universal RNA Extraction Kit (Takara, Shiga, Japan). Reverse transcription and real-time PCR (qRT-PCR) were performed with PrimeScript^TM^ RT Master Mix (Perfect Real Time, Takara) and SYBR^®^ Premix Ex Taq^TM^ II (Tli RNaseH Plus, Takara) following the manufacturer’s instructions. The gene-specific primers are shown in Supplementary Table S3. The relative expression level of mRNA was examined as the inverse log of the delta CT and normalized to the reference gene, β-actin.

### Cell-Cycle Analyses

Following siRNA transfection and 8 Gy γ-ray radiation treatment, the cells were grown in an incubator with 5% CO_2_ at 37°C for 24 h and then collected, fixed in 70% cold ethanol, and stored at −20°C overnight. The cell pellets were washed twice with 1 × phosphate-buffered saline and centrifuged at 1000 × *g* and 4°C for 10 min, following by staining with a cell cycle kit (BD Biosciences, Franklin Lakes, NJ, United States) according to the manufacturer’s instructions. The total cellular DNA content was analyzed with a flow cytometer (Beckman, Brea, CA, United States) by acquiring data for at least 10,000 events.

### Gene Set Enrichment Analysis (GSEA)

Gene set enrichment analysis (GSEA 3.0)^1^ was used to explore potential KEGG pathway and GO analysis within the Molecular Signatures Database (MSigDB 6.0) of c2 (curated gene sets) and c5 (GO gene sets). Setting *ARL14* gene expression levels as population phenotypes in GSEA, we analyzed gene expression omics predictions and assessed related pathways in lung adenocarcinoma. A nominal *P*-value <0.05 and false discovery rate (FDR) <0.25 of the enrichment gene sets in the analysis were considered statistically significant. The theory and process of GSEA have been described previously (Subramanian et al., 2005).

### Statistical Analysis

All data were obtained from 3 to 5 independent experiments and presented as the means ± SE. Statistical analyses were analyzed with Student’s *t*-tests using SPSS 19.0 software (SPSS, Inc., Chicago, IL, United States). *P* < 0.05 was considered to be statistically significant difference between treatment groups.

## Results

### *ARL14* Is a Candidate Prognostic Factor for Lung Adenocarcinoma

**FIGURE 1:**
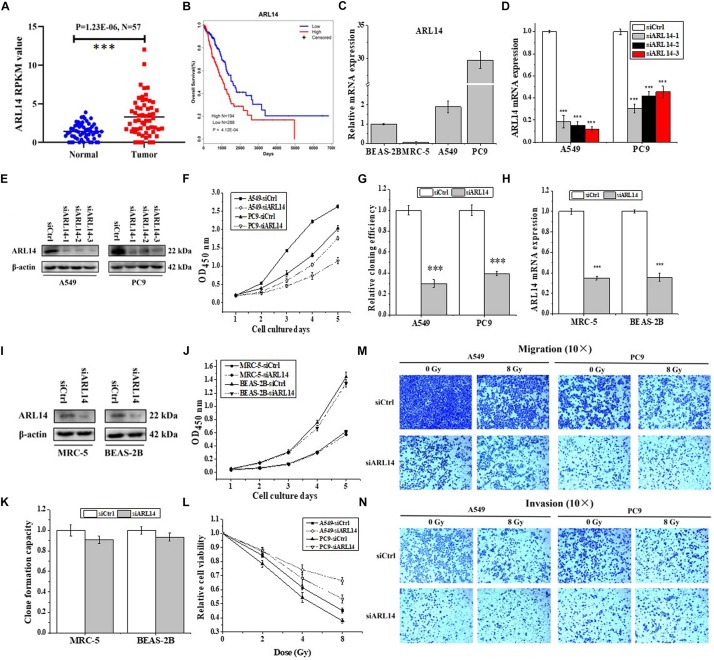
*ARL14* is a potential prognostic biomarker for lung adenocarcinoma and contributes to proliferation, radiosensitivity, migration, and invasion of lung adenocarcinoma cells. **(A)** The expression levels of *ARL14* mRNA in lung adenocarcinoma samples and matched normal control tissues (*N* = 57) in the TCGA cohort. **(B)** Kaplan–Meier analysis of the correlation between *ARL14* level and overall survival of lung adenocarcinoma patients with high (*N* = 194) and low (*N* = 288) *ARL14* expression in the TCGA cohort. Cut-off value for evaluation of *ARL14* mRNA level was the mean expression of 482 lung adenocarcinoma samples. **(C)** The expression levels of *ARL14* mRNA in BEAS-2B, MRC-5, A549, and PC9 cells. **(D,E)** The expressions of *ARL14* mRNA **(D)** and protein **(E)** were reduced in A549 and PC9 cells transfected with *ARL14* siRNAs for 48 h. **(F,G)** Silencing of *ARL14* decreased proliferation **(F)** and cloning efficiency **(G)** of A549 and PC9 cells significantly. **(H,I)** The expressions of *ARL14* mRNA **(H)** and protein **(I)** in MRC-5 and BEAS-2B cells transfected with *ARL14* siRNAs for 48 h. **(J,K)** Silencing of *ARL14* had no significant influence on proliferation **(J)** and cloning efficiency **(K)** of MRC-5 and BEAS-2B cells. **(L)** The dose responses of the viability of A549 and PC9 cells with or without *s*iARL14 transfection. **(M,N)** The migration and invasion activities of A549 and PC9 cells with or without *ARL14* siRNA transfection. After siRNA transfection, cells were irradiated with 8 Gy γ-rays. ^∗∗∗^*P* < 0.001 compared with the corresponding control.

Analyzing the data of lung adenocarcinoma samples and matched normal control tissues obtained from The Cancer Genome Atlas (TCGA) online resource, we found that the mRNA expression of *ARL14* in lung adenocarcinoma samples was significantly higher than that in the matched normal tissues (Figure 1A, *N* = 57, fold change = 2.3, *P* = 1.23E-06), and the expression of *ARL14* in adenocarcinoma had a very strong negative correlation with the overall survival of those lung cancer patients (Figure 1B, *N* = 482, *P* = 4.12E-04). In comparison with the mean level, the lower level of *ARL14* mRNA corresponds to the higher survival. Our measurement further confirmed that the expression levels of *ARL14* in lung adenocarcinoma cells (A549 and PC9) were higher than that in normal lung cells (BEAS-2B and MRC-5) (Figure 1C), which is consistent with the results obtained from TCGA cohort. Therefore, *ARL14* might become a candidate prognostic factor of lung adenocarcinoma development and is worthy of further investigation of its function.

### Knockdown of *ARL14* Suppresses Proliferation, Migration, and Invasion of Lung Adenocarcinoma Cells

To investigate the functions of *ARL14* in lung adenocarcinoma cells, *ARL14* gene expression in A549 and PC9 cells was interfered with siRNA and the silencing effect was detected by qRT-PCR and Western blot assays. As shown in Figures 1D,E, siARL14-1 (hereafter called siARL14) had the most effective efficiency in knock-down *ARL14* expression and thus was applied for the following studies. It was found that siARL14 significantly inhibited cell proliferation (Figure 1F) and cell colony formation ability (Figure 1G) for both A549 and PC9 tumor cells. In addition, siARL14 also effectively suppressed the expression of *ARL14* in normal lung cells MRC-5 and BEAS-2B (Figures 1H,I), however, it had no influence on the proliferation and cloning efficiency of both normal lung cells (Figures 1J,K). These results suggest that *ARL14* may have some special function in cancer cells, which could influence the outcome of radiotherapy. To confirm this hypothesis, we studied the influence of siARL14 on radiation responses of lung adenocarcinoma cells. Results showed that cell proliferations of A549 and PC9 were decreased by γ-ray irradiation in a dose dependent manner, but this decrease was effectively weakened when *ARL14* was knocked down in these cells (Figure 1L). In addition, radiation reduced the abilities of migration and invasion of A549 and PC9 cells but it had no obvious influence on the migration and invasion of siARL14-transfected cells although siARL14 itself suppressed cell migration and invasiveness (Figures 1M,N).

### Knockdown of *ARL14* Induces Lung Adenocarcinoma Cells to Dormancy

**FIGURE 2:**
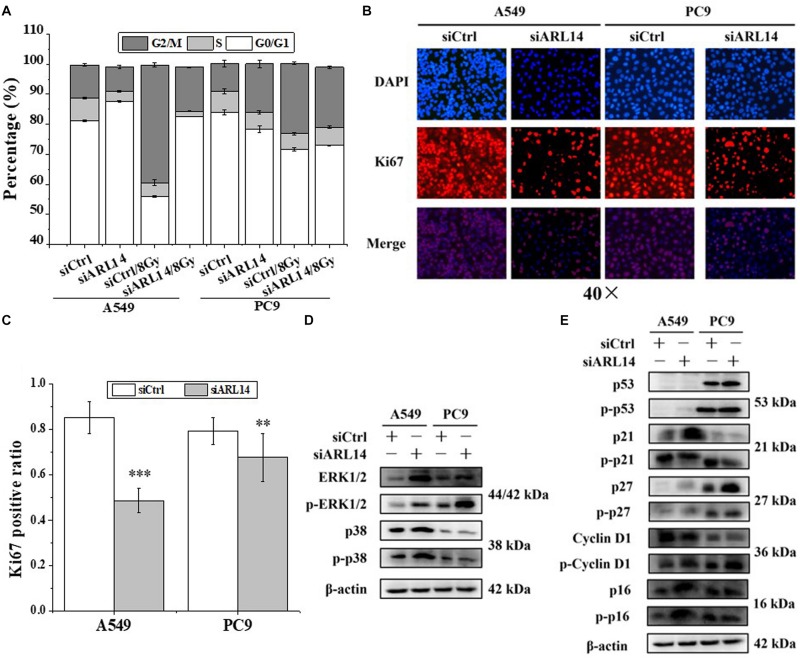
Down-regulation of *ARL14* expression induced quiescence of lung adenocarcinoma cells in G0/G1 phase and inhibited cell proliferation. **(A)** Effects of *ARL14* siRNA on cell cycle distribution and radiation-induced G2/M phase arrest of A549 and PC9 cells. **(B,C)** Ki67 positive ratio in A549 and PC9 cell population transfected with *ARL14* siRNA. **(D,E)** The proteins and their phosphorylation levels of ERK and p38 **(D)**, p16, cyclin D1, p27, p21, and p53 **(E)** in A549 and PC9 cell population transfected with *ARL14* siRNA and its negative control. ^∗∗^*P* < 0.01 and ^∗∗∗^*P* < 0.001 compared with the corresponding control.

Uncontrolled proliferation is a well-established hallmark of cancer cells. Nearly all human cancers have deregulated control in cell-cycle progress. G0 phase is an important check-point when cells decide to begin proliferation or remain quiescence. We monitored the cell cycle distribution by flow cytometer at 24 h after irradiation and found that A549 and PC9 cells were arrested in G2/M phase in accompany with a reduction of cells in G1 phase (Figure 2A and Supplementary Table S4). When the cells were transfected with siARL14, radiation-induced G2/M arrest was effectively released, especially for A549 cells. In fact, silencing of *ARL14* expression resulted in G0/G1 phase accumulation of A549 cells (Figure 2A).

Ki67 protein is an indicator of cell proliferation (Bruno and Darzynkiewicz, 1992). Our immunofluorescence experimental results showed that about 80% cells in the logarithmic growth population of A549 and PC9 had positive Ki67 expression, but when the cells were interfered with siARL14, the ratios of Ki67-positive cells were obviously decreased in both cell lines (Figures 2B,C), indicating cell proliferation ability was attenuated by siARL14.

Imbalance of extracellular signal-regulated kinase (ERK)/p38 signaling activities has also been suggested to determine carcinoma cell proliferation or dormancy (Aguirre-Ghiso et al., 2001, 2003; Ranganathan et al., 2006). We wonder whether ERK and p38 signaling pathways are regulated by *ARL14* in lung adenocarcinoma cells. It was found that when *ARL14* in A549 and PC9 cells was silenced, both total protein and its phosphorylation level of ERK1/2 were increased and p-p38 protein was activated (Figure 2D). A high level of ERK1/2 activity contributes to the promotion of cell proliferation (Aguirre-Ghiso et al., 2001, 2003; Lents et al., 2002; Ranganathan et al., 2006; Chambard et al., 2007). But here silencing *ARL14* caused a distinct decreased cell proliferation, indicating that the ERK signaling pathway is blocked by knockdown of *ARL14* expression, which resulted in the accumulation of ERK and its phosphorylation. To verifying this, we examined whether p21 and cyclin D1 were affected by siARL14. Results showed that when *ARL14* expressions in A549 and PC9 cells were silenced, the expressions of cyclin D1 protein and its phosphorylation level were both increased. However, the p21 protein and its phosphorylation level were upregulated in A549 cells but downregulated in PC9 cells (Figure 2E). Because the cell dormancy after siARL14 transfection was observed in both A549 and PC9 cells according to above Ki67 immunofluorescence assay, we predicted that other cell cycle associated factors may also play important roles in siARL14-induced cell cycle arrest and cell dormancy, such as p16 (Barkan et al., 2008; Sosa et al., 2011), p27 (Barkan et al., 2008; Cackowski et al., 2017; Fluegen et al., 2017; Liu et al., 2018), and p53 (Nagayama et al., 2000; Sosa et al., 2011; Dai et al., 2016). Indeed, our further assay demonstrated that, after siARL14 transfection, both p16, p27 and p53 protein and their phosphorylation levels were upregulated in PC9 cells, and p16 and p27 were upregulated in A549 cells that had abnormal status of p53 (Figure 2E).

### *CIDEC* Is a Downstream Gene of *ARL14*

**FIGURE 3:**
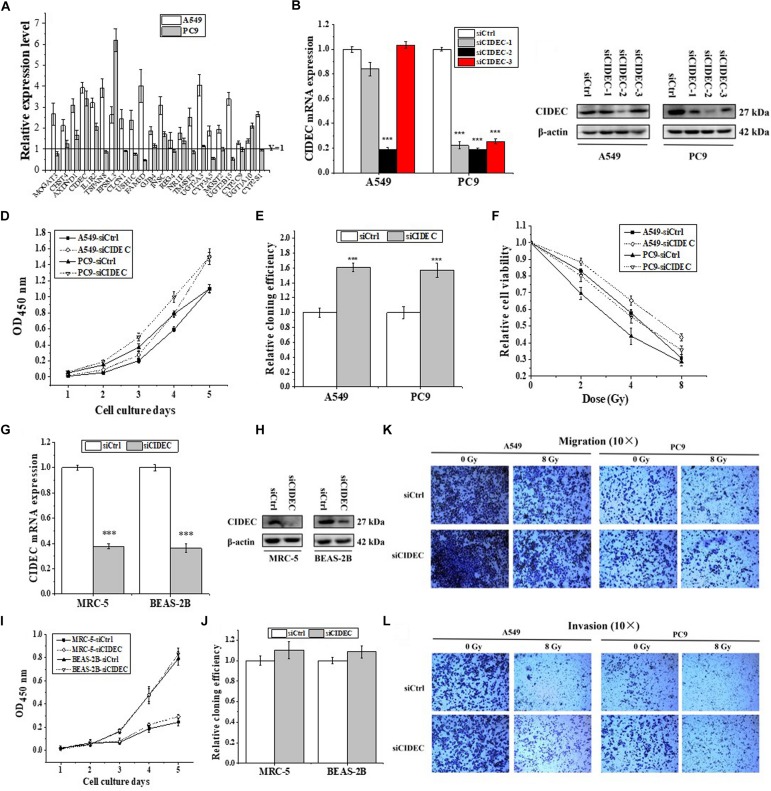
*CIDEC* is a downstream gene of *ARL14* and contributes to proliferation, radiosensitivity, migration and invasion of lung adenocarcinoma cells. **(A)** The candidate genes negatively correlated with *ARL14* analyzed from GESA database and their relative expression levels of mRNA were measured in A549 and PC9 cells transfected with *ARL14* siRNA. **(B,C)** The expressions of *CIDEC* mRNA **(B)** and protein **(C)** in A549 and PC9 cells transfected with *CIDEC* siRNAs for 48 h. **(D–F)** Silencing of *CIDEC* increased proliferation **(D)**, cloning efficiency **(E)**, and radioresistance **(F)** of A549 and PC9 cells. **(G,H)** The expressions of *CIDEC* mRNA **(G)** and protein **(H)** were reduced in MRC-5 and BEASA-2B cells transfected with *CIDEC* siRNAs for 48 h. **(I,J)** Silencing of *CIDEC* had no significant influence on proliferation **(I)** and cloning efficiency **(J)** of MRC-5 and BEAS-2B cells. **(K,L)** The migration and invasion activities of A549 and PC9 cells with or without *CIDEC* siRNA transfection. After siRNA transfection, cells were irradiated with 8 Gy γ-rays. ^∗∗^*P* < 0.01 and ^∗∗∗^*P* < 0.001 compared with the corresponding control.

Seldom study has evaluated the transcriptional regulation of *ARL14* in human cancers. GSEA is a useful tool to reveal the corresponding pathway and regulation mechanism of specific genes (Li et al., 2018), particularly those with unknown functions (Liu et al., 2016). Therefore, the GSEA of 594 RNA-seq data of lung adenocarcinoma from TCGA was performed to gain the insights of interaction networks of *ARL14*. The results suggested that 5850 genes are co-expressed with *ARL14* (Supplementary Data Sheet S1) and the pathways of both positively and negatively correlated with *ARL14* are mostly related to metabolism and immune system (Supplementary Tables S5, S6). Because the absolute magnitude of the correlation index of genes involved in the pathways negatively correlated with *ARL14* were much lower than 0.2 (*P* < 0.05), we focused only on the pathways positively correlated with *ARL14* and examined the mRNA expressions of 22 top-ranking protein encoding genes (Supplementary Data Sheet S1). It was found that only the expressions of *AXDND1*, *CIDEC*, *IL1R2*, *EPS8L3*, and *INSC* genes were significant increased (fold-change > 1.5) in both A549 and PC9 cells after *ARL14* silencing (Figure 3A), indicating that these gene may be downstream of *ARL14*. Figure 3A also showed that *CIDEC* and *EPS8L3* had the highest expressions and the biggest changes in both cell lines, since the function of EPS8L3 protein is still unknown, we focused on the relationship between *ARL14* and *CIDEC*, which has a correlation coefficient of 0.7756 (Supplementary Data Sheet S1).

To further determine the relationship between *ARL14* and *CIDEC*, the function of *CIDEC* in tumor proliferation was then detected. We transfected A549 and PC9 cells with siRNAs against *CIDEC* and the silencing effect were evaluated by qRT-PCR and Western blot assays (Figures 3B,C). The sequence siCIDEC2 (hereafter named siCIDEC) had the most effective efficiency in silencing *CIDEC* gene and thus applied for further experiments. It was found that siCIDEC significantly increased cell proliferation, clone formation and radiation resistance of A549 and PC9 cells (Figures 3D–F). Interestingly, although siCIDEC also reduced the expressions of *CIDEC* gene and protein in both MRC-5 and BEAS-2B cells (Figures 3G,H), it had no significant influence in cell proliferation and clone formation of these normal cells (Figures 3I,J). In addition, silencing of *CIDEC* expression could increase cell migration and invasion and further partly recovered radiation-reduced metastasis ability of both A549 and PC9 cells (Figures 3K,L). Accordingly, *CIDEC* has opposite roles in cell proliferation and migration to *ALR14* in lung adenocarcinoma cells.

**FIGURE 4:**
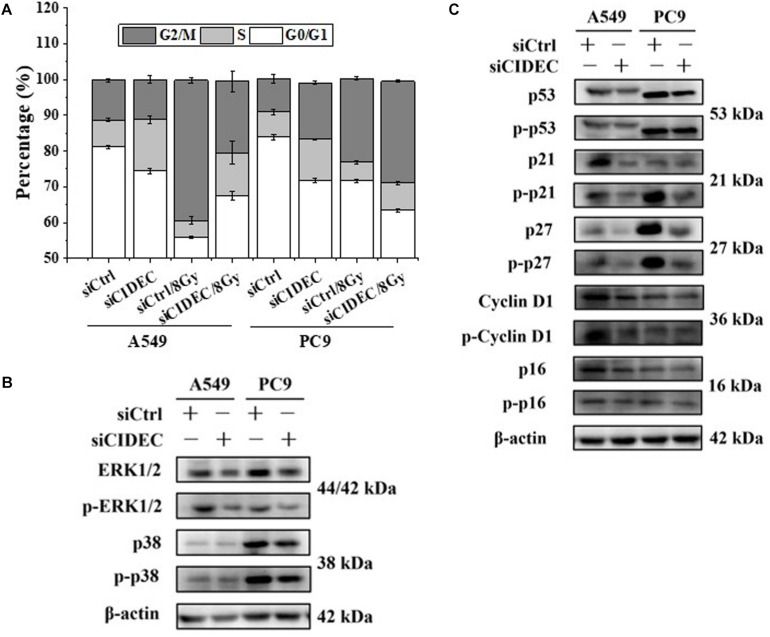
Down-regulation of *CIDEC* expression contributes to the distribution of cell cycle and the expression of cell cycle-related proteins in A549 and PC9 cells. **(A)** Effects of *CIDEC* siRNA on the cell cycle distribution and radiation-induced G2/M phase arrest of A549 and PC9 cells. **(B,C)** The proteins and their phosphorylation levels of ERK and p38 **(B)**, p16, cyclin D1, p27, p21, and p53 **(C)** in A549 and PC9 cell population transfected with *CIDEC* siRNA and its negative control. After siRNA transfection, cells were irradiated with 8 Gy γ-rays.

In addition, cell cycle analysis revealed that silencing of *CIDEC* attenuated the accumulation of cell cycle arrested at G2/M phase of irradiated A549 cells but increased the accumulation of PC9 cells arrested at S-phase (Figure 4A and Supplementary Table S4). Western blot assay shows that the protein and phosphorylation levels of p38 and ERK1/2 and their downstream proteins p16, p21, p27, p53, and cyclin D1 were all down-regulated in A549 and PC9 cells after siCIDEC transfection (Figures 4B,C), which also had a conversed pattern in comparison with that in *ARL14* silencing cells.

## Discussion

*ARL14* is located on human chromosome 13q14.2, a region closely related to several cancers including lung cancer (Ninomiya et al., 2013; Du et al., 2018) and involved in multidrug resistance in cancer treatment (Litviakov et al., 2016). The *ARL14* gene encodes a protein member of the Ras super-family composed of 196 amino acids which is involved in apoptotic signaling and several regulatory pathways (Kahn et al., 1992), indicating its usefulness as a marker for predicting tumor progression and prognosis. We found that *ARL14* had significantly different levels between lung adenocarcinoma samples and matched normal control tissues as well between lung adenocarcinoma cells and normal lung cells, and *ARL14* level is associated with the prognosis of lung adenocarcinoma. Moreover, silencing *ARL14* inhibited the proliferation, migration and invasion of lung adenocarcinoma cells but it had no influence on the proliferation of normal lung cells. Therefore, *ARL14* may be applied as an ideal prognostic biomarker and therapeutic target of lung adenocarcinoma.

Our pilot study showed that, when the lung adenocarcinoma A549 cells were transferred with a lentiviral expression vector of *ARL14* gene, the cell cloning efficiency became very low (<5%) and the cell proliferation was almost total inhibited, indicating that the silencing *ARL14* by lentivirus causes growth arrest and dormancy of lung adenocarcinoma cells. Therefore, this study just transiently transferred siARL14 into lung adenocarcinoma cells and normal lung cells to knock-down *ARL14* expression and found that the percentage of G0/G1-phase cells was increased and the protein level of Ki67 was downregulated in *ARL14* knockdown lung adenocarcinoma cells.

*CIDEC*, a member of the cell-death-inducing DFF45-like effectors family (Liang et al., 2003), is located on human chromosome 3p25, a region associated with a high frequency of loss of heterozygosity in a wide range of tumor tissues. This region plays an important role in the pathogenesis of lung cancer (Graziano et al., 1991). However, few studies of *CIDEC* expression in cancer cells have been reported. Min et al. (2011) found that *CIDEC* expression was decreased in hepatocellular carcinoma tissue compared with its adjacent normal tissues, and overexpression of *CIDEC* inhibited the proliferation of SMMC-7721 cells. In agreement with those studies, our results showed that *CIDEC* silencing promoted cell proliferation, while *ARL14* silencing inhibited proliferation and upregulated *CIDEC* expression in lung adenocarcinoma cells. Moreover, *CIDEC* and *ARL14* had opposite effects on the expressions of proliferation-related proteins and the migration and invasion capacities of lung adenocarcinoma cells. These results supply an evidence that *CIDEC* is downstream of *ARL14* and has antagonistic effect on the biological function of *ARL14*.

Overall, our results demonstrate that silencing *ARL14* can block ERK1/2 and p38 signaling and stimulates its downstream gene *CIDEC* expression, which further activates their downstream effectors of p16, p21, p27, p53, and cyclin D1, resulting in cell cycle arrest of the lung cancer cells. These findings should have implication in identifying the predictive biomarker and treatment targets for lung adenocarcinoma.

## Data Availability Statement

The datasets generated for this study are available on request to the corresponding author.

## Author Contributions

FG, DY, and CS: conceptualization. FG, JuZ, HZ, CW, LZ, and JiZ: methodology. FG, DY, and JuZ: formal analysis. FG and DY: investigation. FG: draft writing. YP and CS: review and editing. FG: visualization. CS: project administration. YP and CS: funding acquisition. CS: supervision.

## Conflict of Interest

The authors declare that the research was conducted in the absence of any commercial or financial relationships that could be construed as a potential conflict of interest.

## Footnotes

1 http://software.broadinstitute.org/gsea/index.jsp
