# Supplementary material for: Silencing of ARL14 Gene Induces Lung Adenocarcinoma Cells to a Dormant State
Source: Front Cell Dev Biol. 2019 Oct 15;7:238. doi: 10.3389/fcell.2019.00238 (PMC6843082; doi:10.3389/fcell.2019.00238)
Supplement: Supplementary file 2 [file Table_1.DOC]

Supplemental for the article:

**Silencing of *ARL14* Gene Induces Lung Adenocarcinoma Cells to a Dormant State**

Fei Guo, Dexiao Yuan, Junling Zhang, Hang Zhang, Chen Wang, Lin Zhu, Jianghong Zhang, Yan Pan* and Chunlin Shao*

(Institute of Radiation Medicine, Fudan University, Shanghai 200032, China)

**Correspondence:**

Prof. Chunlin Shao, [clshao@shmu.edu.cn;](mailto:clshao@shmu.edu.cn;)

Dr. Yan Pan, [swallowpan@fudan.edu.cn](mailto:swallowpan@fudan.edu.cn)

**Supplemental Table 1** The target sequences of transient transfection of siRNA

| Gene | Target sequence (5' to 3') | |
| --- | --- | --- |
| ARL14 | siARL14-1 | GGATGTTGGAGGACAGGAA |
|  | siARL14-2 | GGCTGTTACTGTGAGAACA |
|  | siARL14-3 | GAGCCACATGAAATCAAGA |
| CIDEC | siCIDEC-1 | GCATCATGGCTTACAGTCT |
|  | siCIDEC-2 | GGGATACAGTGTTCATGGT |
|  | siCIDEC-3 | CACTGTCCCTCTCCCATAA |

**Supplemental Table 2** List of antibodies in this study

| Antibody | Company | Dilution rate |
| --- | --- | --- |
| Anti-ARL14-[antibody](https://www.abcam.cn/arl15-antibody-ab106441.html) | Abcam | 1:1000 |
| Anti-p21 antibody | Abcam | 1:1000 |
| Anti-p38 antibody | Abcam | 1:1000 |
| Anti-p27 KIP 1 (p-S10) antibody | Abcam | 1:1000 |
| Anti-β Actin antibody | Abcam | 1:3000 |
| Anti-p44/42 MAPK (Erk1/2) antibody | CST | 1:1000 |
| Anti-phospho-p44/42 MAPK (Erk1/2)  (Thr202/Tyr204) antibody | CST | 1:1000 |
| Anti-p27 KIP 1 antibody | CST | 1:1000 |
| Anti-phospho-p38 MAPK (Thr180/Tyr182) antibody | CST | 1:1000 |
| Anti-p53 antibody | CST | 1:1000 |
| Anti-phospho-p53 (Ser15) antibody | CST | 1:1000 |
| Anti-ki67 antibody | CST | 1;500 |
| Anti-phospho-p21(Ser146) antibody | Santa Cruz | 1:200 |
| Anti-p16 antibody | Santa Cruz | 1:200 |
| Anti-cyclin D1 antibody | Santa Cruz | 1:200 |
| Anti-phospho-p16(Ser152) antibody | SAB | 1:1000 |
| Anti-phospho-cyclin D1(Thr288) antibody | SAB | 1:1000 |
| Anti-CIDEC antibody | Proteintech | 1:1000 |
| Alexa Fluor® 594 goat anti-mouse IgG (H+L) | Thermo Fisher Scientific | 1:1000 |

**Supplemental Table 3** The primer sequences of qRT-PCR

| Gene | Primer sequence (5' to 3') |
| --- | --- |
| ARL14 | F: AAATCCGCAAACCAAACAAGC  R: TTCCAACTCGATCATTTCCACAT |
| MOGAT3 | F: CCAACCACTTCCAAAACCTTGC  R: TGCCCGGTTCCTTATCCACT |
| CHST4 | F: CCTGCTGTTTCTGGTTTCCCA  R: TGCCCCACAAAAGAAGAGCC |
| AXDND1 | F: TATGGTGGATCGTTCAAAACTCC  R: GGCCGCATAGGTCAGAGAAA |
| CIDEC | F: AAGTCCCTTAGCCTTCTCTACC  R: CCTTCCTCACGCTTCGATCC |
| IL1R2 | F: ATGTTGCGCTTGTACGTGTTG  R: CCCGCTTGTAATGCCTCCC |
| TSPAN8 | F: ACTTCTTGTTCTGGCTATGTGG  R: CACAGCAACGTAGGAGCTAGA |
| EPS8L3 | F: AGCCATTTACTTGCACCGGAA  R: CTCCCCTGCTTGCATGTCAT |
| CLCN1 | F: ATGGAGCAATCCCGGTCAC  R: AGGGCATATACTGGTACTGGG |
| USH1C | F: TTCCGGCATAAGGTGGATTTTC  R: GTACATTCGCAGCACATCATAGA |
| FAM3D | F: CTGCCCAGCCAACTACTTTG  R: CTCCCGTGGTTCCATTCAC |
| GJB4 | F: CTGCTGAGTGGCGTGAACAA  R: CACACGAAAGATGAACACCACA |
| INSC | F: TCAAACGGGGTTGGGTCATTA  R: CTCAATCTCGCCCATTTCCTT |
| REG4 | F: CTGCTCCTATTGCTGAGCTG  R: GGACTTGTGGTAAAACCATCCAG |
| NR1I2 | F: AAGCCCAGTGTCAACGCAG  R: GGGTCTTCCGGGTGATCTC |
| TM4SF4 | F: AGGAAGCGGTGTCTTGATGAT  R: GGAGGTGAACATCGCAAATCG |
| UGT2A3 | F: GCCTTCGTTAATTGACTACAGGA  R: GTTGATAAGCCTGGCAAGACAT |
| CYP3A5 | F: AATGTTTTGTCCTATCGTCAGGG  R: AGACCTTCGATTTGTGAAGACAG |
| MGST2 | F: TCGGCCTGTCAGCAAAGTTAT  R: TGTTGTGCCCGAAATACTCTCT |
| UGT2B15 | F: CCAACCAATGAAGCCCCTG  R: GTTGTGAGCTGCGACTCGAA |
| CYP2C9 | F: GCCTGAAACCCATAGTGGTG  R: GGGGCTGCTCAAAATCTTGATG |
| UGT1A10 | F: GCCCCGTTCCTTTATGTGTGT  R: ATCTTCCAGAGTGTACGAGGTT |
| CYP2S1 | F: GCGCTGTATTCAGGGCTCAT  R: CTTCCAGCATCGCTACGGTT |
| β-actin | F: TGACGTGGACATCCGCAAAG  RCTGGAAGGTGGACAGCGAGG |
| F: forward; R: reverse. | |

**Supplemental Table 4 Effect of silencing ARL14 or CIDEC on cell cycle distribution of A549 and PC9 Cells**

| Cell types | Treatment |  | G0/G1 (%) | S (%) | G2/M (%) |
| --- | --- | --- | --- | --- | --- |
| A549 | siCtrl | 0 Gy | 81.14±0.38 | 7.56±0.37 | 11.03±0.43 |
|  |  | 8 Gy | 55.95±0.31 | 4.61±1.06 | 39.18±0.78 |
|  | siARL14 | 0 Gy | 87.55±0.31 | 3.44±0.27 | 8.15±0.50 |
|  |  | 8 Gy | 82.50±0.06 | 1.79±0.17 | 14.75±0.20 |
|  | siCIDEC | 0 Gy | 74.40±0.80 | 14.34±0.95 | 11.26±0.95 |
|  |  | 8 Gy | 67.56±1.12 | 11.98±3.2 | 19.99±2.91 |
| PC9 | siCtrl | 0 Gy | 83.88±0.83 | 7.01±0.82 | 9.32±1.18 |
|  |  | 8 Gy | 71.65±0.46 | 5.19±0.49 | 23.40±0.43 |
|  | siARL14 | 0 Gy | 78.43±1.16 | 5.57±0.53 | 16.10±1.33 |
|  |  | 8 Gy | 72.91±0.16 | 6.15±0.49 | 20.00±0.42 |
|  | siCIDEC | 0 Gy | 71.68±0.65 | 11.57±0.12 | 15.77±0.51 |
|  |  | 8 Gy | 63.46±0.49 | 7.58±0.40 | 28.49.±0.37 |

**Supplemental Table 5 The top 20 pathway** positively associated with ARL14

| NAME | SIZE | ES | NES |
| --- | --- | --- | --- |
| METABOLISM OF XENOBIOTICS BY CYTOCHROME P450 | 19 | 0.684044 | 1.72579 |
| RETINOL METABOLISM | 22 | 0.687672 | 1.695786 |
| CHEMICAL CARCINOGENESIS | 25 | 0.615896 | 1.692684 |
| DRUG METABOLISM - CYTOCHROME P450 | 18 | 0.665014 | 1.669578 |
| PROTEIN DIGESTION AND ABSORPTION | 14 | 0.663768 | 1.660089 |
| GLYCOSPHINGOLIPID BIOSYNTHESIS - LACTO AND NEOLACTO SERIES | 9 | 0.803821 | 1.63292 |
| STEROID HORMONE BIOSYNTHESIS | 13 | 0.700746 | 1.620956 |
| PORPHYRIN AND CHLOROPHYLL METABOLISM | 12 | 0.658694 | 1.57776 |
| FAT DIGESTION AND ABSORPTION | 9 | 0.697467 | 1.568355 |
| PENTOSE AND GLUCURONATE INTERCONVERSIONS | 10 | 0.701002 | 1.537585 |
| ASCORBATE AND ALDARATE METABOLISM | 8 | 0.716819 | 1.497908 |
| DRUG METABOLISM - OTHER ENZYMES | 15 | 0.576699 | 1.44836 |
| MATURITY ONSET DIABETES OF THE YOUNG | 8 | 0.738006 | 1.394286 |
| MUCIN TYPE O-GLYCAN BIOSYNTHESIS | 15 | 0.581261 | 1.386584 |
| SEROTONERGIC SYNAPSE | 22 | 0.497269 | 1.378667 |
| BILE SECRETION | 21 | 0.521586 | 1.36906 |
| GLYCEROLIPID METABOLISM | 14 | 0.557617 | 1.363561 |
| NEUROACTIVE LIGAND-RECEPTOR INTERACTION | 14 | 0.551088 | 1.333962 |
| STARCH AND SUCROSE METABOLISM | 18 | 0.527694 | 1.295477 |
| ENDOCRINE AND OTHER FACTOR-REGULATED CALCIUM REABSORPTION | 10 | 0.590441 | 1.265083 |
| ES: enrichmentscore; NES: normalizeden-richmentscore. | | | |

**Supplemental Table 6** The top 20 pathway negatively associated with ARL14

| NAME | SIZE | ES | NES |
| --- | --- | --- | --- |
| GLYCOSAMINOGLYCAN BIOSYNTHESIS - CHONDROITIN SULFATE / DERMATAN SULFATE | 7 | -0.73291 | -1.41713 |
| STAPHYLOCOCCUS AUREUS INFECTION | 14 | -0.72437 | -1.24778 |
| RNA DEGRADATION | 8 | -0.54992 | -1.17851 |
| ALLOGRAFT REJECTION | 8 | -0.72363 | -1.14164 |
| GRAFT-VERSUS-HOST DISEASE | 8 | -0.72363 | -1.14164 |
| ASTHMA | 8 | -0.72363 | -1.1397 |
| HTLV-I INFECTION | 33 | -0.37757 | -1.00183 |
| INTESTINAL IMMUNE NETWORK FOR IGA PRODUCTION | 12 | -0.55427 | -0.99231 |
| FATTY ACID METABOLISM | 16 | -0.39543 | -0.9765 |
| ANTIGEN PROCESSING AND PRESENTATION | 12 | -0.56167 | -0.97618 |
| PROGESTERONE-MEDIATED OOCYTE MATURATION | 14 | -0.36172 | -0.9156 |
| SPLICEOSOME | 11 | -0.4199 | -0.9117 |
| PRION DISEASES | 9 | -0.44323 | -0.86671 |
| GLYCOSPHINGOLIPID BIOSYNTHESIS - GANGLIO SERIES | 7 | -0.39665 | -0.85177 |
| SYSTEMIC LUPUS ERYTHEMATOSUS | 17 | -0.43039 | -0.84133 |
| AUTOIMMUNE THYROID DISEASE | 11 | -0.44731 | -0.82833 |
| OLFACTORY TRANSDUCTION | 7 | -0.3383 | -0.82199 |
| VIRAL MYOCARDITIS | 18 | -0.34208 | -0.80467 |
| TYPE I DIABETES MELLITUS | 9 | -0.47277 | -0.79655 |
| RHEUMATOID ARTHRITIS | 22 | -0.34122 | -0.79245 |
| ES: enrichmentscore; NES: normalizeden-richmentscore. | | | |
